# Loss of epithelial FAM20A in mice causes amelogenesis imperfecta, tooth eruption delay and gingival overgrowth

**DOI:** 10.1038/ijos.2016.14

**Published:** 2016-06-03

**Authors:** Li-Li Li, Pei-Hong Liu, Xiao-Hua Xie, Su Ma, Chao Liu, Li Chen, Chun-Lin Qin

**Affiliations:** 1 Harbin Medical University School of Stomatology, Harbin, China; 2 Department of Stomatology, the Second Affiliated Hospital of Harbin Medical University, Harbin, China; 3 Department of Biomedical Sciences and Center for Craniofacial Research and Diagnosis, Texas A&M University Baylor College of Dentistry, Dallas, USA; 4 Longjiang Scholar Laboratory, the First Affiliated Hospital of Harbin Medical University, Harbin, China

**Keywords:** conditional knock out mice, enamel, FAM20A, gingival overgrowth, tooth eruption

## Abstract

FAM20A has been studied to a very limited extent. Mutations in human *FAM20A* cause amelogenesis imperfecta, gingival fibromatosis and kidney problems. It would be desirable to systemically analyse the expression of FAM20A in dental tissues and to assess the pathological changes when this molecule is specifically nullified in individual tissues. Recently, we generated mice with a *Fam20A*-floxed allele containing the beta-galactosidase reporter gene. We analysed FAM20A expression in dental tissues using X-Gal staining, immunohistochemistry and *in situ* hybridization, which showed that the ameloblasts in the mouse mandibular first molar began to express FAM20A at 1 day after birth, and the reduced enamel epithelium in erupting molars expressed a significant level of FAM20A. By breeding *K14-Cre* mice with *Fam20A*^*flox/flox*^ mice, we created *K14-Cre;Fam20A*^*flox/flox*^ (conditional knock out, cKO) mice, in which *Fam20A* was inactivated in the epithelium. We analysed the dental tissues of cKO mice using X-ray radiography, histology and immunohistochemistry. The molar enamel matrix in cKO mice was much thinner than normal and was often separated from the dentinoenamel junction. The *Fam20A*-deficient ameloblasts were non-polarized and disorganized and were detached from the enamel matrix. The enamel abnormality in cKO mice was consistent with the diagnosis of amelogenesis imperfecta. The levels of enamelin and matrix metalloproteinase 20 were lower in the ameloblasts and enamel of cKO mice than the normal mice. The cKO mice had remarkable delays in the eruption of molars and hyperplasia of the gingival epithelium. The findings emphasize the essential roles of FAM20A in the development of dental and oral tissues.

## Introduction

In mammals, the FAM20 family (family with sequence similarity 20) consists of the three following members: FAM20A, FAM20B and FAM20C.^1^ Recent findings have led to great excitement about this small family of proteins, which have endoplasmic reticulum-entry signal sequences responsible for directing proteins into the secretory pathway and which appear to be involved in the post-translational modifications of secretory proteins.

FAM20C is a kinase that phosphorylates many extracellular matrix proteins involved in biomineralization and other biological processes.^2, 3^ *FAM20C* is ubiquitously expressed,^1, 4, 5^ and mutations in the human FAM20C gene cause Raine syndrome, an autosomal recessive disorder that demonstrates a broad spectrum of clinical manifestations.^6, 7, 8, 9, 10^ *Fam20C*-deficient mice developed hypophosphataemic rickets, along with severe dental defects.^11, 12^

FAM20B is a kinase that catalyses the attachment of phosphate to xylose, which is a step essential to the assembly of glycosaminoglycans during the synthesis of proteoglycans.^13^ Defects in *FAM20B* have not been associated with human genetic disease. Constitutive inactivation of *Fam20B* in mice was embryonically lethal,^14^ whereas conditional deletion of *Fam20B* in the rodent dental epithelium led to supernumerary incisors.^15^

FAM20A was originally observed in the lungs and liver, and it displayed obvious differential expression in haematopoietic cells undergoing myeloid differentiation.^1^ FAM20A is believed to be a pseudokinase that cannot independently catalyse the attachment of phosphate to other molecules, but it can form a functional complex with FAM20C and can enhance the capacity of the latter to phosphorylate extracellular proteins in their secretory pathways.^16^ *In vitro* studies have shown that FAM20A potentiates the kinase activity of FAM20C and promotes the FAM20C-catalysed attachment of phosphate to enamel matrix proteins, such as enamelin.^16^ The *in vitro* findings that FAM20A stimulates FAM20C-dependent phosphorylation of certain enamel matrix proteins have provided some basic information for understanding the molecular pathogenesis underlying the enamel defects associated with the mutations in, and deletion of, the *FAM20A* gene. The mutations in human *FAM20A* cause amelogenesis imperfecta with gingival fibromatosis syndrome (AIGFS, OMIM #614253) and enamel renal syndrome (ERS, OMIM #204690).^17, 18, 19, 20, 21^ Enamel defects have been described in mice with constitutive loss of *Fam20A*.^14^ Related dental anomalies in human subjects include generalized hypoplastic enamel, tooth eruption delay, intrapulpal calcification and fibrotic enlargement of the gingiva.^20^ Compared with FAM20C, FAM20A has been studied only to a limited extent, and there is a need to profile systematically the expression of FAM20A in dental tissues and to analyse the pathological and molecular changes that occur when this molecule is specifically ablated in individual tissues or cells. Recently, we created *Fam20A*-floxed mice, in which the floxed allele contained the beta-galactosidase (LacZ) reporter gene, allowing us to reveal the expression of FAM20A with X-Gal staining and to inactivate this molecule in the tissues or cells of interest. In this study, we specifically ablated *Fam20A* from mouse epithelium by breeding *Fam20A*^*flox/flox*^ mice with *K14-cre* mice to create *K14-Cre;Fam20A*^*flox/flox*^ (*K14-Cre;Fam20A*^*Δ/Δ*^ *or* conditional knock out (cKO)) mice. The cKO mice manifested enamel defects, tooth eruption delay and gingival epithelium overgrowth.

## Materials and methods

### Generation of *Fam20A-*floxed mice

**Figure 1:**
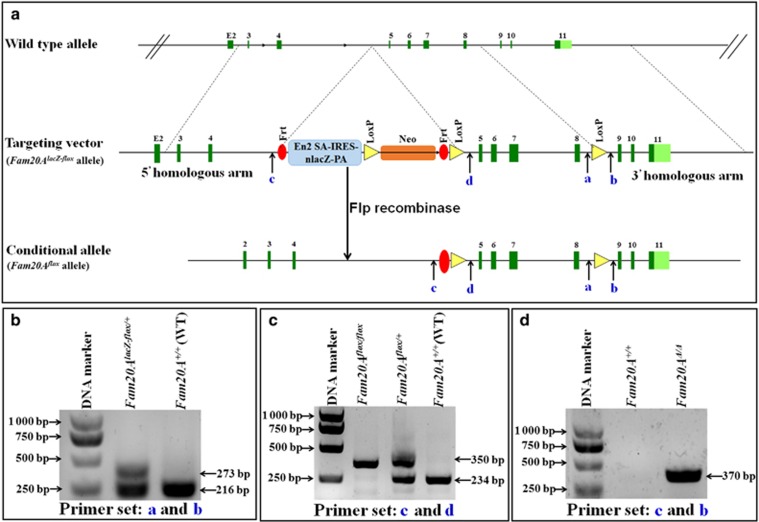
**Targeting vector for creating *Fam20A*-mutant mice and genotyping strategy**. (**a**) The mouse *Fam20A* gene contains 11 exons (green boxes). Intron 4 is the largest among the 10 introns. In the targeting vector, an IRES-lacZ-Neo cassette flanked by two flippase recognition target (FRT) sites (red ovals) was inserted into intron 4, and one LoxP site (yellow triangle) was placed into intron 8, which is also relatively large. Recombination after Flp recombinase scission removed the IRES-lacZ-Neo cassette from the *Fam20A*^*lacZ-flox*^ allele and produced the conditional allele *Fam20A*^*flox*^, in which the region of exons 5 through 8 was flanked by two LoxP sites (yellow triangles). (**b**) We used the primer set of **a** and **b** to distinguish the *Fam20A*^*lacZ-flox*^ allele from the WT allele; PCR with these primers produced a 273-bp fragment for the mutant allele and 216-bp fragment for WT allele. (**c**) The primer set of **c** and **d** was used to identify the *Fam20A*^*flox*^ allele after the IRES-lacZ-Neo cassette was removed by Flp recombinase. Note that the use of primers **c** and **d** was not expected to generate any PCR products for the *Fam20A*^*lacZ-flox*^ allele. (**d**) The primer set of **c** and **b** was used to identify the *Fam20A-*ablated (*Fam20*^*Δ*^) allele in the *K14-Cre;Fam20A*^*flox/+*^ or the *K14-Cre;Fam20A*^*flox/flox*^ mice; PCR with these primers did not produce any fragment for the *Fam20A-*floxed (*Fam20A*^*flox/+*^ and *Fam20A*^*flox/flox*^) or WT alleles. PCR, polymerase chain reaction; WT, wild type.

Mice with a *Fam20A*-floxed allele were generated by Biocytogen (Beijing, China). In the targeting vector used to create the floxed allele, exogenous elements were inserted into intron 4 and intron 8 of the mouse *Fam20A* gene; the region from exon 5 through exon 8 was flanked by two loxP sequences (Figure 1a). Both intron 4 and intron 8 are sufficiently large, and insertion of loxP elements into these two introns is not expected to interfere with mRNA splicing. When planning the targeting strategy, we calculated that removal of exon 5 though exon 8 would result in a frame shift for protein translation and create a truncated FAM20A with 247 amino acids, which could be subject to nonsense-mediated decay. In addition, the amino acid sequence encoded by the region of exon 5 through exon 8 in the *Fam20A* gene is highly conserved among species, and it also shows strong similarity to the amino acid sequence of the kinase domain in the *Fam20C* and *Fam20B* genes. Therefore, ablating the region from exon 5 through exon 8 in the *Fam20A* gene should result in functional loss of the FAM20A protein.

Intron 4 of the targeting vector also contained an IRES-lacZ-Neo cassette flanked by two FRT sites so that a conditional allele could be generated after treatment with flippase (Flp) recombinase (Figure 1a). Before introducing Flp into the mice, the LacZ reporter gene encoding beta-galactosidase was present, allowing for the utilization of X-Gal staining to visualize the expression pattern of FAM20A. After the introduction of Flp recombinase, the IRES-lacZ-Neo cassette was removed, leaving the region of exons 5–8 flanked by two loxP sites (Figure 1a). We designated the mice (founders and their progenies) with one allele of *Fam20A* containing the IRES-lacZ-Neo cassette as “*Fam20A*^*lacZ-flox/+*^ mice” and referred to the mice with one allele of *Fam20A* that was floxed by two loxP elements but that contained no IRES-lacZ-Neo cassette as “*Fam20A*^*flox/+*^ mice.” The *Fam20A*^*lacZ-flox/+*^ mice were used for X-Gal staining to reveal the expression profile of FAM20A. The *Fam20A*^*flox/+*^ mice were inbred to create *Fam20A*^*flox/flox*^ mice.

DNA extracted from the mouse tails was analysed by polymerase chain reaction (PCR) for genotyping to identify the *Fam20A*^*lacZ-flox/+*^ allele using the following set of primers: forward, 5′-GTCATTGAAGGAGTTGCCACTGTC-3′ (“a” in Figure 1a); and reverse, 5′-CAGGTAGCCTCAAACATGCAACAT-3′ (“b” in Figure 1a). PCR with these primers gave rise to a product of 273 base pairs (bp) for the *Fam20A*^*lacZ-flox*^ allele and a 216-bp fragment for the wild-type allele (Figure 1b).

### Generation of *K14-Cre;Fam20A*^*flox/flox*^

First, we mated *K14-Cre* mice, which express Cre recombinase in the epithelium of the oral mucosa approximately 10.5 days post coitum (d.p.c.),^22, 23^ with *Fam20A*^*flox/flox*^ mice to create *K14-Cre;Fam20A*^*flox/+*^mice. Then, the *K14-Cre;Fam20A*^*flox/+*^ mice were bred with *Fam20A*^*flox/flox*^ mice to generate *K14-Cre;Fam20A*^*flox/flox*^ (that is, *K14-Cre;Fam20A*^*Δ/Δ*^) mice, which we refer to as cKO mice in this report. DNA samples from mouse tails were analysed by PCR for genotyping. A set of primers specific for the *Cre* transgene was adopted to identify the *Cre* transgene. The sequences of primers specific for the *Cre* transgene were: forward, 5′-ATTTGCCTGCATTACCGGTC-3′ and reverse, 5′-ATCAACGTTTTCTTTTCGG-3′. PCR with the *Cre*-specific primers produced a 350-bp fragment when the DNA samples from mice expressing the *Cre* recombinase were used as the template and produced no PCR product for the wild-type allele (data not shown).

One pair of primers with the forward sequence of 5′-GAAACTTTGCAGTCCTTGTTCCC-3′ (“c” in Figure 1a, located in intron 4 and upstream of the first Frt element in the targeting vector) and the reverse sequence of 5′-GCACTATCAATGCCAAGTTTCC-3′ (“d” in Figure 1a, located in intron 4 and downstream of the second LoxP site) was used to distinguish the *Fam20A-*floxed (*Fam20A*^*flox/+*^ or *Fam20A*^*flox/flox*^) alleles from the wild-type alleles; the use of these primers generated a 350-bp fragment for the *Fam20A-*floxed alleles in the *Fam20A*^*flox/+*^ or *Fam20A*^*flox/flox*^ mice and a 234-bp fragment for the wild-type alleles (Figure 1c).

Another set of primers was designed to distinguish the *Fam20A-*ablated (*Fam20A*^*Δ*^) allele in the *K14-Cre;Fam20A*^*flox/+*^ or the *K14-Cre;Fam20A*^*flox/flox*^ mice from the *Fam20A-*floxed (*Fam20A*^*flox/+*^ and *Fam20A*^*flox/flox*^) or wild-type alleles. The sequences of primers used to identify the *Fam20A-*ablated allele were: forward, 5′-GAAACTTTGCAGTCCTTGTTCCC-3′ (“c” in Figure 1a); and reverse, 5′-CAGGTAGCCTCAAACATGCAACAT-3′ (“b” in Figure 1a); this set of primers generated a 370-bp fragment for the *Fam20A-*null alleles and produced no PCR band for the *Fam20A-*floxed or wild-type alleles (Figure 1d).

The use of mice was approved by the Experimental Animal Ethics Committee of Harbin Medical University, China, and was in accordance with the recommendations in the Guide for Care and Use of Laboratory Animals published by the USA National Institutes of Health. All the mice analysed in this study were fed a normal (hard) diet after weaning. For each analysis at each time point, specimens from at least three mice were evaluated.

### Analyses at the gross level

In this investigation, we observed the incisors, molars and gingiva of the mice at different ages. The mice were weighed every week from birth until postnatal 56 days to monitor animal growth. The average values of the body weight calculated from three mice in each age group were used to generate growth curves.

### X-Gal staining

For X-Gal staining, the mandibles dissected from the *Fam20A*^*lacZ-flox/+*^ and wild-type (control) mice at the ages of 13.5, 14.5, 15.5 and 17.5 days post coitum and 0, 1, 5, 7 and 11 days after birth, respectively, were fixed in 4% ice-cold paraformaldehyde for 1 h on a shaker and were washed with phosphate-buffered saline (PBS) solution for 45 min. Then, the mandibles were decalcified in 15% ethylene diaminetetraacetic acid (EDTA) solution (pH 7.4) at 4 °C for 1–7 days, depending on the ages of the animals. The samples were processed for sucrose infiltration, and 10-μm serial frozen sections were prepared with a cryostat microtome. The frozen sections were incubated in X-Gal solution (Gold Biotechnology, St Louis, MO, USA) for 24 h at 37 °C in darkness and were counterstained with Sirius Red and observed under a microscope.

### Haematoxylin and eosin and immunohistochemistry staining

For haematoxylin and eosin (H&E) and immunohistochemistry (IHC) staining, the mouse mandibles were fixed with 4% paraformaldehyde in PBS solution at 4 °C overnight and then were decalcified in 15% EDTA solution at 4 °C for 0–7 days, depending on the ages of the animals. The samples were processed for paraffin embedding, and serial sections of 5 μm in thickness were cut for H&E and IHC analyses.

The polyclonal antibody against FAM20A (Abcam, Cambridge, MA, USA) was used at a dilution of 1:200. The polyclonal antibodies against enamelin (ENAM), ameloblastin (AMBN) and matrix metalloproteinase 20 (MMP20) were purchased from Santa Cruz (Santa Cruz, CA, USA) and were used at dilutions of 1:200, 1:800 and 1:100, respectively. The specimens of the normal and cKO mice from the same litters were stained in the same batch of experiments to ensure better comparability. Normal rabbit serum at the same concentrations as the polyclonal antibodies was used to replace the polyclonal anti-FAM20A, anti-ENAM and anti-AMBN antibodies and served as a negative control. Goat serum was used to replace the anti-MMP20 antibody, acting as a negative control for this polyclonal antibody. All of the IHC experiments were performed using the ABC kit for polyclonal antibodies (Vector Laboratories, Burlingame, CA, USA). The 3,3′-diaminobenzidine (DAB) kit (Vector Laboratories, Burlingame, CA, USA) was used for colour development, according to the manufacturer's instructions. The sections were counterstained with methyl green. Each set of the IHC experiments was repeated at least three times.

### *In situ* hybridization

For *in situ* hybridization (ISH) analyses, diethylpyrocarbonate-treated solutions were used for processing the mouse mandibles and for the hybridization experiments to ensure RNase-free conditions. We generated a 681-bp RNA probe that was complementary to the mouse FAM20A mRNA, using the same method as we previously described.^11, 24^ Briefly, a primer set with 5′-ACAATTCAACCTTACCTCCTTGG-3′ (in exon 2 of the mouse *Fam20A* gene) for forward and 5′-CTTTTCCTGACAGCGAGTAGG-3′ (in exon 8) for reverse was used to generate a cDNA fragment using PCR, with total RNA extracted from the molars of 16.5-day-old mice as the template. Next, the PCR products were cloned into the pCRII-TOPO vector (Promega, Madison, WI, USA) and were transformed into competent *Escherichia coli*. The plasmid DNA was isolated from *E. coli* and sequenced. Digoxigenin-labelled single-stranded RNA probes were synthesized and labelled with digoxigenin using an RNA labelling kit (Roche, Indianapolis, IN, USA), and the DIG-labelled RNA probes were detected by enzyme-linked immunoassay with a specific anti-DIG-AP antibody conjugate, as we previously described.^11, 24^ These RNA probes were used to detect FAM20A mRNA in the paraffin sections containing tissues from the mouse mandible.

### Plain X-ray and micro-computed tomography

Mandibles of normal and cKO mice at the ages of 1, 2, 4 and 6 weeks after birth were examined by a Faxitron X-ray Radiography System (MX-20; Faxitron X-ray, Wheeling, IL, USA) at a voltage of 26 kVp and an exposure time of 8.5 s. The first molars dissected from the mandibles of the 4- and 6-week-old mice were radiographed by a micro-computed tomography (μCT) system (μCT35; Scanco Medical AG, Bassersdorf, Switzerland) with a 12-μm voxel size using the following parameters: 114 mA (current), 70 kVp (voltage) and 300 μs (exposure time). The scanning process lasted for approximately 1 h per sample and generated approximately 600 images for each sample.

## Results

### Expression of FAM20A in the dental tissues of mice

Because the development of the mouse first molar resembles and is usually used to represent human teeth, the current study focused on the expression of FAM20A in the mandibular first molar. We were particularly interested in the presence or absence of FAM20A signals in ameloblasts at different developmental stages of the mouse molar because the cKO mice had enamel defects with relatively normal dentin. In addition, representative images from the X-Gal staining of mandibular incisor regions are presented to demonstrate the expression of FAM20A in incisor ameloblasts at different stages.

**Figure 2:**
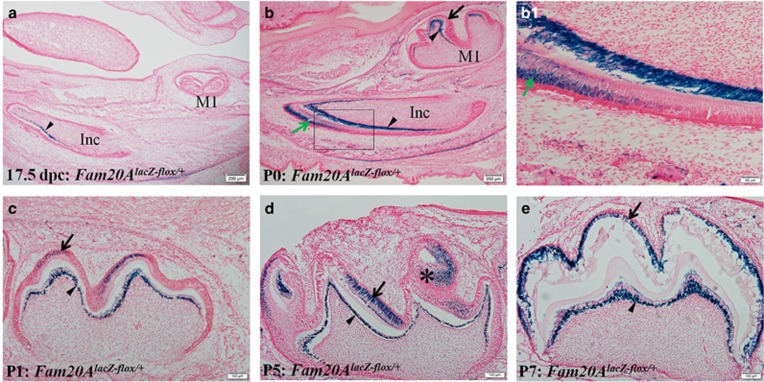

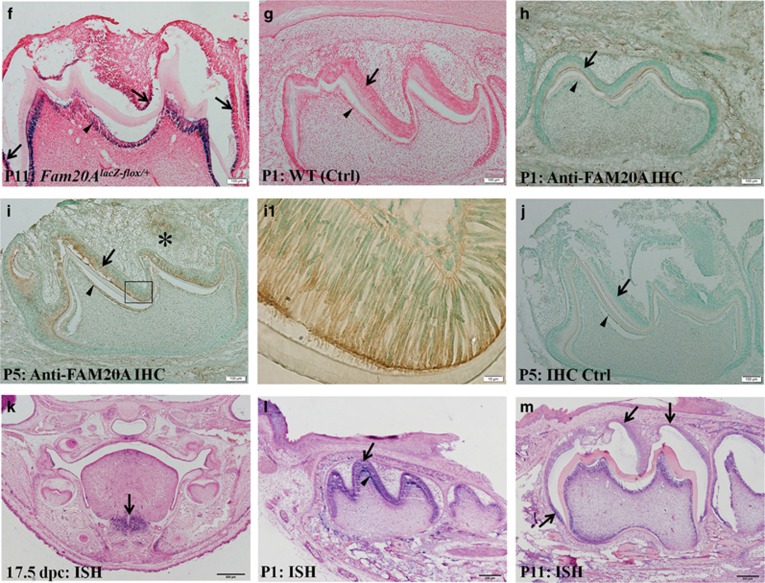
**Expression of FAM20A in the mouse dental tissues**. (**a**) X-Gal staining revealed the presence of FAM20A signals in the odontoblasts (arrowhead) of the mandibular incisors and its total absence from the first molars (M1) of the *Fam20A*^*lacZ-flox/+*^ mice at 17.5 days post coitum. (**b**) At postnatal day 0 (P0), FAM20A signals were observed in the odontoblasts (arrowhead) but not in the ameloblasts (arrow) of the mandibular first molar (M1); at this stage, FAM20A signals were also observed in the secretory stage ameloblasts (green arrow) in the incisor; (**b1**) higher magnification view of the box area in **b**. (**c**) At P1, the FAM20A signals in the odontoblasts (arrowhead) were stronger than in the ameloblasts (arrow). (**d**) At P5, the FAM20A signals in the ameloblasts (arrow) of some regions were stronger than in the odontoblasts (arrowhead); relatively strong FAM20A signals were also observed in some cells of the stellate reticulum (asterisk). (**e**) At P7, the intensity of FAM20A signals in the ameloblasts (arrow) was similar to that in the odontoblasts (arrowhead). (**f**) At P11, FAM20A signals were present in the reduced enamel epithelium (arrows) and odontoblasts (arrowhead) in the *Fam20A*^*lacZ-flox/+*^ mice. (**g**) Wild-type mice did not show any X-Gal-positive staining. (**h**) Immunohistochemistry showed weak anti-FAM20A immunoreactivity in the ameloblasts (arrow) and the odontoblasts (arrowhead) of the mandibular first molar at P1. (**i**) At P5, strong anti-FAM20A immunoreactivity was observed in the first molar ameloblasts (arrow), odontoblasts (arrowhead) and stellate reticulum (asterisk); (**i1**) higher magnification view of the box area in **i** revealed that, in the ameloblasts, FAM20A protein was almost exclusively located in the cytoplasm on the enamel matrix side of the nuclei. (**j**) Immunohistochemistry staining with the first antibody-free control showed no FAM20A signal in the ameloblasts (arrow) or odontoblasts (arrowhead). (**k**) *In situ* hybridization analyses showed that, at 17.5 days post coitum, FAM20A mRNA was not observed in any of the tooth germs but was observed in the tongue root region (arrow). (**l**) At P1, FAM20A mRNA was observed in the ameloblasts (arrow) and in the odontoblasts (arrowhead) of the mandibular first molar. (**m**) At P11, FAM20A mRNA was present in the reduced enamel epithelium (arrows) and odontoblasts (arrowhead) of the first molar. (**a**–**g**) X-Gal staining of the mouse mandible; (**h**–**j**) anti-FAM20A IHC staining of the first molar region in the mouse mandible; (**k**–**m**) *in situ* hybridization staining of mouse head and jaw bones. Scale bars, 200 μm (**a** and **b**); 50 μm (**b1**); 10 μm (**i1**); 500 μm (**k**); 200 μm (**l** and **m**); 100 μm (all other images). P0, P1, P5, P7 and P11 are abbreviations of postnatal day 0, 1, 5, 7 and 11, respectively. Ctrl, control; d.p.c., days post coitum; IHC, immunohistochemistry; Inc, incisor; ISH, *in situ* hybridization.

X-Gal staining was not observed in the molars of wild-type mice, confirming the high specificity of this approach. X-Gal staining showed that, at 17.5 days post coitum, FAM20A signals were present in the odontoblasts (arrowhead) of the mandibular incisor but not in any cells of the first molar in the *Fam20A*^*lacZ-flox/+*^ mice (Figure 2a). At postnatal day 0 (birth), FAM20A signals were absent from the ameloblasts but were present in the odontoblasts in the mandibular first molars of *Fam20A*^*lacZ-flox/+*^ mice (Figure 2b); at this time point, FAM20A signals were also observed in both the ameloblasts and odontoblasts of the mandibular incisor; in the incisor, FAM20A was localized in the secretory stage ameloblasts (Figure 2b and 2b1). At postnatal day 1, although FAM20A was observed in both the ameloblasts and odontoblasts in the mandibular first molar, the X-Gal staining of the former cells was weaker than previously (Figure 2c). At postnatal day 1, FAM20A was primarily observed in the ameloblasts of the cusp tips in the mandibular first molar. At postnatal day 5, the intensity of FAM20A signals in the molar ameloblasts became stronger than at postnatal day 1 and was comparable to that in the odontoblasts of the mandibular first molar (Figure 2d). At postnatal day 5, significant FAM20A signals were also observed in some cells of the stellate reticulum (Figure 2d, asterisk). At postnatal day 7, the FAM20A signals in the molar ameloblasts were similar to those in the odontoblasts; intensive staining was observed in nearly all of the ameloblasts and odontoblasts (Figure 2e). At postnatal day 11, when the mandibular first molar was in its eruptive phase, a significant level of FAM20A was observed in the reduced enamel epithelium (Figure 2f, arrows). In the mandibular incisors of 5- and 7-day-old *Fam20A*^*lacZ-flox/+*^ mice, X-Gal staining showed that FAM20A signals were localized in the secretory stage ameloblasts and maturation stage ameloblasts (Supplementary Figure 1a and 1b). X-Gal staining also showed positive signals for FAM20A in the gingiva of postnatal 28-day-old mice (Supplementary Figure 1c). Throughout the observation period, the beta-galactosidase activity was either totally absent or barely visible in the cells of the dental follicles and periodontal ligaments. The X-Gal staining analyses demonstrated the absence of FAM20A signals in the ameloblasts and odontoblasts of the molars and incisors in the *Fam20A*^*lacZ-flox/+*^ mice at 13.5, 14.5 and 15.5 days post coitum (data not shown). These findings indicated that the ameloblasts in the first molar of the mouse mandible began to express FAM20A at 1 day after birth, and the stellate reticulum and reduced enamel epithelium expressed significant levels of FAM20A.

IHC analyses (Figure 2h–2j) showed that, at postnatal day 1, the anti-FAM20A immunoreactivity was weakly positive in the ameloblasts and odontoblasts of the mandibular first molar (Figure 2h). At day 5 after birth, FAM20A was observed in the ameloblasts (arrow), stellate reticulum (asterisk) and odontoblasts (arrowhead) of the mandibular first molar (Figure 2i and 2i1), in agreement with the X-Gal staining results. In the ameloblasts, FAM20A protein was uniformly located in the cytoplasm on the enamel matrix side of the nuclei (that is, towards the dentinoenamel junction direction). In the odontoblasts, FAM20A protein was primarily located in the cytoplasm on the predentin side of the nuclei. It should be noted that there are currently only a few anti-FAM20A antibodies commercially available, and we tried two of them in this study. Although the anti-FAM20A antibody described in this report recognized FAM20A, its titre and specificity were low; thus, the IHC analyses in this investigation were not as sensitive as the X-Gal staining in assessing the expression of FAM20A. Nevertheless, the IHC experiments were necessary because such approaches could assess the actual localization of FAM20A protein in the cells, constituting an advantage over the X-Gal staining used to monitor the expression of this molecule in the *Fam20A*^*lacZ-flox/+*^ mice, which contained beta-galactosidase in the nuclei of FAM20A-expressing cells.

ISH analyses demonstrated that, at 17.5 days post coitum, FAM20A mRNA was present in the lingual root region of the embryo but was absent from the ameloblasts or the odontoblasts of any tooth germs (Figure 2k). At postnatal day 1, FAM20A mRNA was detected in both the ameloblasts and odontoblasts of the mandibular first molar; the signals in the former were weaker than in the latter (Figure 2l), consistent with the X-Gal staining results. At postnatal day 11, when the mandibular first molar was in its eruptive phase, and the outer and inner enamel epithelia were fused to become reduced enamel epithelium, a relatively strong level of FAM20A mRNA was observed in the reduced enamel epithelium (Figure 2m, arrows). The ISH data lent further support to the conclusion drawn from the X-Gal staining that the ameloblasts in the mouse mandibular first molar started to express FAM20A at postnatal day 1 and that the reduced enamel epithelium expressed a significant level of FAM20A.

### Overall assessment of *Fam20A*-mutant mice

The body sizes, teeth and gingivae of the *Fam20A*^*flox/+*^ mice, *Fam20A*^*flox/flox*^ mice or *K14-Cre;Fam20A*^*flox/+*^ mice were same as the age-matched wild-type mice, indicating that insertion of the loxP elements or *Fam20A* haploinsufficiency did not cause developmental abnormalities. Because the *Fam20A*^*flox/flox*^ mice were completely normal, we used as normal control (Ctrl) mice from the same litters as the cKO mice created during the crossbreeding regime. Utilizing *Fam20A*^*flox/flox*^ littermates of cKO mice as normal controls not only reduced the number of utilized mice, but it also prevented potential variances that might result from comparing animals from different litters.

**Figure 3:**
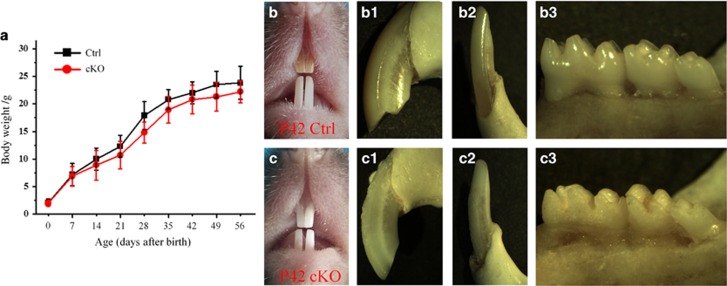
**Assessment of normal and cKO mice at the gross level**. (**a**) Growth curve of mice from postnatal day 0 to 56. The 28- and 35-day-old cKO mice were smaller than the control mice, whereas the body weights of the 56-day-old cKO were close to those of the normal (control) mice. (**b**) Gross photograph of incisors in the 42-day-old control mice; (**b1**) the upper incisor (with gingiva removed) in 42-day-old control mice; (**b2**) the lower incisor (with gingiva removed) in 42-day-old control mice; (**b3**) the mandibular molars (with gingiva removed) in 42-day-old control mice. (**c**) Gross photograph of incisors in the 42-day-old cKO mice; (**c1**) the upper incisor (with gingiva removed) in 42-day-old cKO mice; (**c2**) the lower incisor (with gingiva removed) in 42-day-old cKO mice; (**c3**) the mandibular molars (with gingiva removed) in 42-day-old cKO mice. Note the differences in the colour and surface smoothness between the teeth of normal and cKO mice. Ctrl, normal control group; cKO, conditional knockout group.

The cKO mice were apparently smaller than the normal mice at postnatal 28 and 35 days. As the mice aged, this difference in body size between the cKO and normal mice became decreasingly remarkable; the body sizes of 56-day-old cKO mice were close to those of the normal control mice at the same age (Figure 3a).

### Enamel defects in cKO mice

The labial sides of the upper and lower incisors of the cKO mice had a chalky-white and opaque appearance, which was in clear contrast to those of the normal mice, which showed a yellow-brown colour and a transparent glossy appearance (Figure 3b and 3b1). Compared with the mandibular molars of the normal mice, the molars of the cKO mice had a rough surface and appeared yellow, likely caused by rapid, abrasive loss of enamel and exposure of the underlining yellow dentin (Figure 3b3 and 3c3).

**Figure 4:**
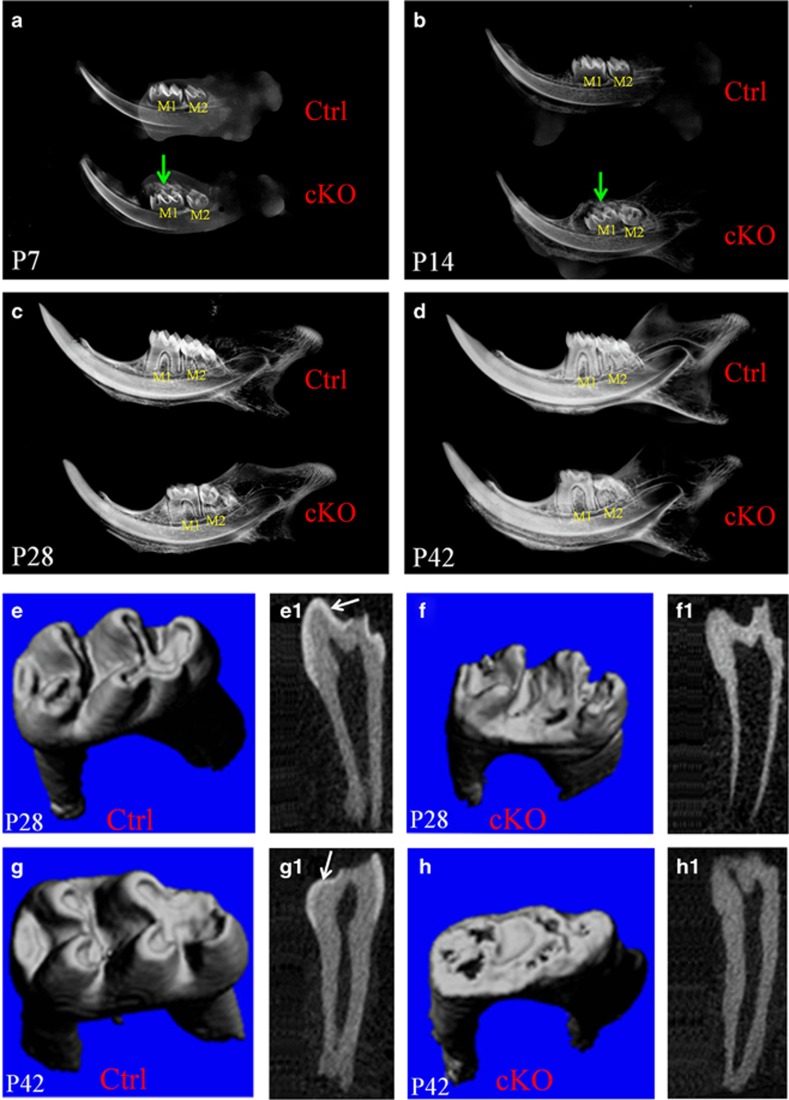
**X-ray radiography analyses**. (**a**–**d**) The plain X-ray radiographic analyses of the mandibles from the 7-day-old (P7), 14-day-old (P14), 28-day-old (P28) and 42-day-old (P42) mice. (**e**–**h**) μCT analyses of mandibular first molars from 28-day-old (P28) and 42-day-old (P42) mice. (**a**) At P7, the mineralized walls of pulp chambers in the mandibular first molars of the cKO mice appeared thinner than in control mice; the first molar in the cKO mice was covered by a large amount of overtopping bone (arrow) on the oral cavity side, whereas no obvious bone tissue was observed on the oral cavity side of the first molar in the normal mice. (**b**) At P14, the pulp chamber walls in cKO mice were thinner compared with control mice, and a significant amount of bone (arrow) was still present between the mandibular first molar and the oral cavity in the cKO mice. (**c**, **d**) In the P28 and P42 cKO mice, the pulp chamber walls were much thinner, and the crowns were shorter than in the control mice. These X-ray observations indicated that the eruption of the mandibular first and second molars in the cKO mice was dramatically delayed. M1, mandibular first molar; M2, mandibular second molar; arrows indicate the bone tissues overlying the oral cavity side of the mandibular first molars. (**e**, **f**) Compared with the control mice, the occlusal surface of the mandibular first molar in the P28 cKO mice was rougher. (**e1**, **f1**) On section views, an enamel layer (arrow) was clearly visible in the molars of the normal mice, whereas no enamel or enamel-like structures could be identified in the molars of the cKO mice. (**g**, **h**) At P42, the occlusal surface of the first molar in the cKO mice was nearly flat, and the tooth crown was much shorter than in the control mice. (**g1**, **h1**) An enamel layer (arrow) was clearly visible in the control mice but not in the cKO mice. **e**, **f**, **g** and **h**: full views; **e1**, **f1**, **g1** and **h1**: longitudinal-section views. cKO, conditional knock out; Ctrl, control; μCT, micro-computed tomography.

Because plain X-ray analyses could not distinguish the enamel layer from the dentin, the radiopaque area on X-ray radiography reflected the whole mineralized walls of pulp chamber and root canals. Plain X-ray examination showed that, at postnatal day 7, the mineralized walls of pulp chambers in the crowns of the mandibular first molars of the cKO mice were thinner than in the normal control mice (Figure 4a), likely due to a reduction in the thickness of the enamel layer. At postnatal day 14, the pulp chamber walls were much thinner in the molar crowns of the cKO mice than in the control mice (Figure 4b). At postnatal day 28 and day 42, the thin wall defects in the mandibular first molars of the cKO mice became even more remarkable (Figure 4c and 4d), which could be attributed to the loss of the hypomineralized enamel layer after the mice started to chew food. In addition, the crowns of the mandibular first molars of the cKO mice were much shorter than in the normal mice. The plain X-ray examination also revealed a dramatic delay in the eruption of the mandibular first and second molars in the cKO mice (see below).

The μCT analyses could clearly distinguish the enamel layer from the dentin in the mandibular first molars of the normal mice, whereas in the mandibular first molars of the cKO mice, μCT radiography did not reveal any structure in the pulp chamber wall with a mineralization level differing from that of the molar dentin (Figure 4e–4h1). These observations indicated that either the mineralization level of the *Fam20A*-deficient enamel was too low to make it distinguishable from the dentin, or perhaps the very thin layer of enamel that formed in the cKO mice was rapidly lost after the tooth began to function. At postnatal day 28, the occlusal surface of the mandibular first molar in the cKO mice appeared rougher than in the normal mice (Figure 4e and 4f). At postnatal day 42, the mandibular first molars in the cKO mice lost nearly all of their cusps and became flattened, in clear contrast to those in the normal mice, which had distinct cusps and a glossy surface (Figure 4g and 4h). The crown of the mandibular first molar in the 42-day-old cKO mice was much shorter than in the normal mice at the same age. The shortened crown of the mandibular first molar in the 42-day-old cKO mice was likely attributed to the rapid loss of coronal hard tissue due to attrition. The mice start to chew food after weaning at approximately 21 days postnatally. The loss of mineralized tissue in the first molar crowns of 28-day-old cKO mice, which had been in occlusion for approximately 7 days, was much less than in the molar crowns of 42-day-old cKO mice, which had been in function for approximately 21 days.

**Figure 5:**
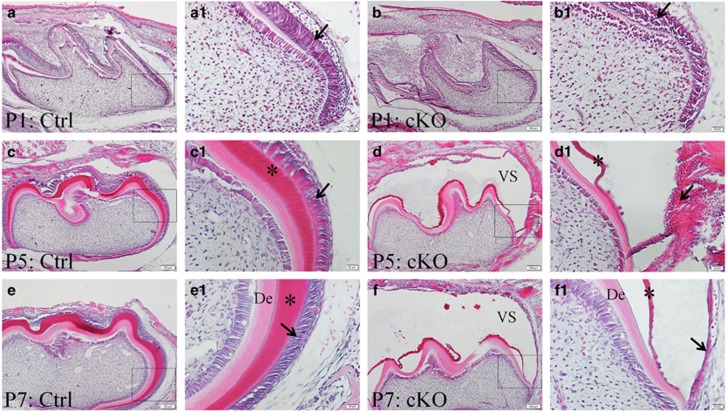
**H&E staining of the mandibular first molars from 1-day-old (P1), 5-day-old (P5) and 7-day-old (P7) mice**. (**a**) H&E staining of the mandibular first molar region from 1-day-old normal (control) mice. (**a1**) Higher magnification view of the box area in **a** showed that the ameloblasts (arrow) in control mice were high columnar, polarized and well aligned. (**b**) H&E staining of the mandibular first molar region from 1-day-old cKO mice. (**b1**) Higher magnification view of the box area in **b** demonstrated that the ameloblasts (arrow) in cKO mice were cuboid, non-polarized and disorganized. (**c**, **c1**) In the 5-day-old control mice, the mandibular first molar formed a significant amount of enamel matrix (asterisk in **c1**), and high columnar ameloblasts (arrow in **c1**) were tightly attached to the enamel matrix. (**d**, **d1**) In the 5-day-old cKO mice, very little enamel matrix was formed, and the thin layer of enamel matrix (asterisk in **d1**) was also often detached from the dentinoenamel junction. The flattened and clustered ameloblasts (arrow in **d1**) seemed to be “pushed” away from the enamel matrix, and the separation of ameloblasts from the enamel matrix in the cKO mice created a void space. (**e**, **e1**) H&E staining of the mandibular first molar region from the 7-day-old control mice showed that the enamel matrix (asterisk in **e1**) beneath the well-developed ameloblasts (arrow in **e1**) was tightly attached to dentin. (**f**, **f1**) The void space between the ameloblasts (arrow in **f1**) and enamel matrix (asterisk in **f1**) became more prominent. The molar dentin was not much different between the control and cKO groups. Scale bars, 100 μm (**a**, **b**, **c**, **d**, **e** and **f**); 20 μm (**a1**, **b1**, **c1**, **d1**, **e1** and **f1**). cKO, conditional knock out; Ctrl, control; De, dentin; H&E, Haematoxylin and eosin; VS, void space.

Histology analyses with H&E staining showed that, at postnatal day 1, the overall morphology of the mandibular first molars in the cKO mice was similar to that of the normal mice (Figure 5a and 5b). Although the ameloblasts in the normal mice were highly columnar, polarized and well organized (Figure 5a1, arrow), those in the cKO mice were low columnar or cuboid, non-polarized and disorganized (Figure 5b1, arrow). At postnatal day 5, the mandibular first molars in the normal mice formed a significant amount of enamel matrix (Figure 5c and 5c1, asterisk in 5c1), whereas those of the cKO mice had very little enamel matrix (Figure 5d and 5d1, asterisk in 5d1). The ameloblast layer in the mandibular first molars of the normal mice was closely attached to the enamel matrix (Figure 5c and 5c1), whereas that in the cKO mice was detached from the enamel matrix (Figure 5d and 5d1). The separation of ameloblasts from the enamel matrix in the cKO mice created a void space (“VS” in Figure 5d), in which a certain amount of liquid seemed to accumulate. The ameloblasts in the mandibular first molars of the cKO mice (arrow in Figure 5d1) were “pushed” away from the enamel matrix and became flattened and clustered, in contrast to the high columnar, well-polarized and well-organized ameloblasts in the normal mice (arrow in Figure 5c1). At postnatal day 7, the void space between the ameloblast mass and enamel matrix in the cKO mice (“VS” in Figure 5f) became more prominent. At either postnatal day 5 or day 7, the dentin in the cKO mouse molar was not much different from that in the normal mice.

**Figure 6:**
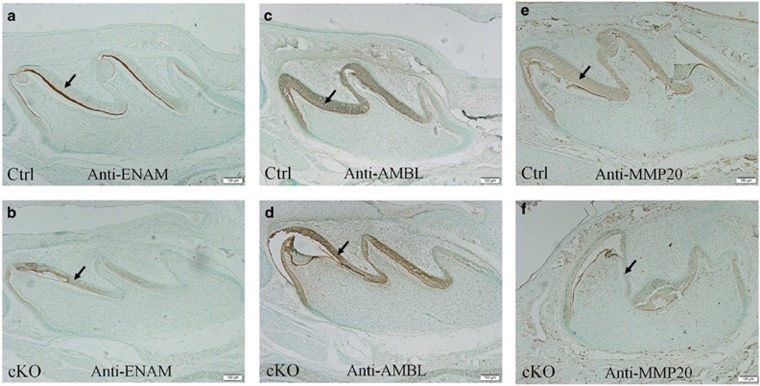
**Immunohistochemistry analyses of ENAM, AMBL and MMP20 in the mandibular first molars from 1-day-old (P1) mice**. (**a**) In the normal (control) mice, ENAM protein was highly concentrated in the region of the ameloblast cytoplasm, close to the basal lamina zone. (**b**) The anti-ENAM signal was remarkably weaker and more diffused in the *Fam20A*-deficient ameloblasts than in the normal cells. (**c**, **d**) The AMBL signal was stronger in the *Fam20A*-null ameloblasts than in the normal mice. (**e**, **f**) The immunoreactivity for MMP20 was weaker in the ameloblasts of cKO mice than in the normal mice. Arrows indicate ameloblasts. Scale bars=100 μm in all the images. cKO, conditional knock out; Ctrl, control.

IHC analyses were performed on the paraffin sections from postnatal day 1 and day 7 mice. The immunostaining differences in ENAM, AMBL and MMP20 between the 7-day-old cKO and normal mice were similar to those observed in the 1-day-old samples. Thus, only the IHC results from the 1-day-old mice are presented in this report. In the normal mice, ENAM protein was highly concentrated in the distal region of the ameloblasts close to the basal lamina zone, although its signal was also observed in the other regions of the ameloblast cytoplasm (Figure 6a). The anti-ENAM signal was remarkably weaker in the *Fam20A*-deficient ameloblasts (Figure 6b) than in the normal cells. The distribution of ENAM in the *Fam20A*-deficient ameloblasts also appeared to be more diffused than in the normal cells. In the 7-day-old normal mice, the enamel matrix close to the basal lamina zone and ameloblasts showed very strong anti-ENAM immunoreactivity, whereas in the 7-day-old cKO mice, the anti-ENAM signal was hardly visible in the enamel matrix (data not shown). The anti-AMBL signal was stronger in the ameloblasts of cKO mice than in the normal mice (Figure 6c and 6d). The immunoreactivity for MMP20 in the ameloblasts of cKO mice was remarkably weaker than in the normal mice (Figure 6e and 6f).

### Tooth eruption delay in cKO mice

**Figure 7:**
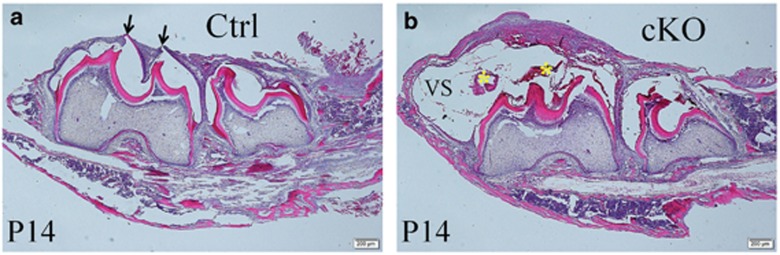
**Haematoxylin and eosin staining of the first and second molar regions in the mandibles from 14-day-old (P14) mice**. (**a**) In the normal (control) mice, the reduced enamel epithelium in the cusp tip region of the mandibular first molar fused with the oral epithelium, and the cusp tips (arrows) were about to emerge in the oral cavity. (**b**) In the cKO mice, there was still a great distance between the crown of the first molar and the oral cavity; the void space (VS) contained tissue/cellular debris (yellow asterisks), which appeared to have fallen into the cavity from the surrounding soft tissues. Scale bars, 200 μm in both images. cKO, conditional knock out; Ctrl, control; VS, void space.

Plain X-ray radiography showed that, at postnatal day 7, the mesial and middle cusps of the mandibular first molars in the normal mice were close to the mucosa of the oral cavity, and there was very little or no bone tissue overlying the oral cavity side of the tooth, whereas the cusps of the mandibular first molars in the cKO mice were far away from the oral cavity, and a large amount of the overtopping bone (arrow in Figure 4a) was covering the tooth. At postnatal day 14, there were still significant amounts of bone present between the mandibular first molar and the oral cavity in the cKO mice (Figure 4b). Histology analyses revealed that the reduced enamel epithelium of the mandibular first molar had fused with the oral mucosa, and the tooth was about to emerge into the oral cavity in the 14-day-old normal mice (Figure 7a). In the 14-day-old cKO mice, there was still a significant distance between the crown of the mandibular first molar and the oral mucosa (Figure 7b). At postnatal day 14, a large void space (“VS” in Figure 7b) was present between the reduced enamel epithelium and the crown of the mandibular first molar in the cKO mice, and the void space contained tissue/cellular debris (asterisks in Figure 7b) that appeared to have fallen off from the surrounding soft tissue. At postnatal day 28, the mandibular second molar in the normal mice had fully emerged in the oral cavity, whereas the mandibular second molar in the cKO mice was still covered by the jawbone on the oral cavity side (Figure 4c). On the bais of our X-ray and histology observations, we estimated that the eruption of the mandibular first molar in the cKO mice was delayed by approximately 10 days.

### Gingival overgrowth in cKO mice

**Figure 8:**
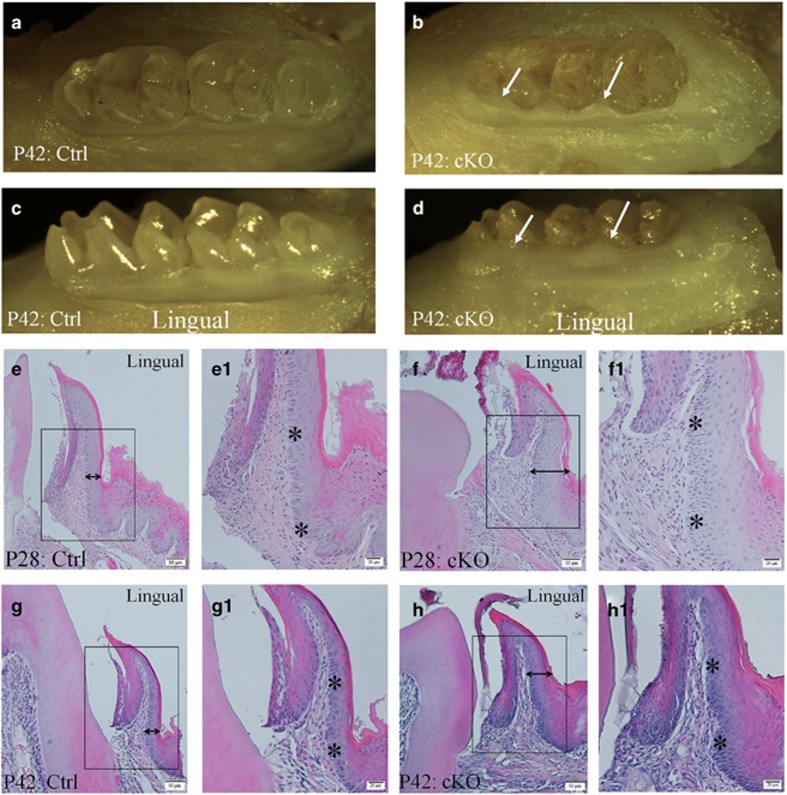
**Gingival overgrowth in the cKO mice**. (**a**) Gross photograph on occlusal view of the mandibular molar region in the 42-day-old (P42) normal (control) mice. (**b**) Gross photograph on occlusal view of the mandibular molar region in the P42 cKO mice; arrows indicate the enlarged gingiva. (**c**) Gross photograph on lingual view of the mandibular molar region in the P42 control mice. (**d**) Gross photograph on lingual view of the mandibular molar region in the P42 cKO mice; arrows indicate the enlarged gingiva. (**e**) H&E staining of a buccal–lingual section from the mandibular first molar region showed the lingual side gingiva in the 28-day-old control mice; double arrow indicates the thickness of the epithelium. (**e1**) Higher magnification view of the box area in **e** revealed a single layer of basal cells (asterisks). (**f**) The epithelium thickness (double arrow) in the lingual side gingiva of P28 cKO mice was greater than in the normal mice. (**f1**) Higher magnification view of the box area in **f** revealed multiple layers of basal cells (asterisks). (**g**) H&E stain image of the lingual side gingiva in P42 normal mice; double arrow indicates the thickness of the epithelium. (**g1**) Higher magnification view of the box area in **g** revealed a single layer of basal cells (asterisks). (**h**) The epithelium thickness (double arrow) in the lingual side gingiva of P42 cKO mice was greater than in the normal mice. (**h1**) Higher magnification view of the box area in **h** revealed multiple layers of basal cells (asterisks). Scale bars, 50 μm (**a**, **b**, **c** and **d**); 20 μm (**a1**, **b1**, **c1** and **d1**). cKO, conditional knock out; Ctrl, control; H&E, Haematoxylin and eosin.

The cKO mice showed noticeable gingival overgrowth, which was more remarkable on the lingual side than on the buccal side. The gingival overgrowth was clearly visible on gross observation (Figure 8a–8d). Histology analyses of H&E-stained sections revealed that the gingival epithelium in the cKO mice was obviously thicker than in the normal mice at postnatal day 28 (Figure 8e and 8f). The basal cells in the gingival epithelium formed a single and uniform layer in the normal mice (asterisks in Figure 8e1), whereas in the cKO mice, the gingival epithelium often had multiple layers of basal cells (asterisks in Figure 8f1). The basal cells or basal cell-like cells in the *Fam20A*-deficient gingival epithelium also appeared misaligned or disorganized. At postnatal day 42 (Figure 8g–8h1), the histological appearance of the hyperplastic gingiva in the cKO mice was similar to that observed in the 28-day-old mice.

## Discussion

FAM20A is a member of a small gene family that includes three proteins, all of which contain endoplasmic reticulum-entry signal peptides that guide molecules into the secretory pathway.^1^ FAM20A is postulated to be localized primarily in the Golgi apparatus, where it is believed to form a complex with FAM20C and to enhance the catalytic activity of the latter in phosphorylating certain secretory proteins.^16^ Deficiency in FAM20A or FAM20C might alter the post-translational modifications of their substrate proteins, leading to pathological changes in tissues in which the proper functions of these kinases or their substrates are essential. The critical roles of FAM20A and FAM20C in the development of dental tissues have been well demonstrated in human and mouse genetic studies, which have shown the associations of *FAM20A* or *FAM20C* deficiencies with inherited dental defects.^6, 7, 8, 9, 10^^,^^14,^^17, 18, 19, 20, 21^

Information about FAM20A and the available tools to study this molecule are limited. Although a previous study showed the expression of FAM20A in ameloblasts and odontoblasts,^21^ there has been a lack of systematic profiling for the expression of FAM20A in dental tissues. Multipronged approaches in the current study showed that the ameloblasts in the first molars of mouse mandibles started to express FAM20A at postnatal day 1, a time point when the morphogenesis of the tooth has completed. Our previous study showed that the FAM20C was expressed in the dental epithelium of the cap-stage enamel organ at approximately 14.5 days post coitum;^4^ the expression of FAM20C in the dental tissue occurred much earlier than that of FAM20A. In addition, FAM20C was also much more broadly expressed than FAM20A.^1^ The differences in the temporospatial distribution between FAM20A and FAM20C suggested that the former might not be always essential to the physiological function of the latter, although the two molecules are postulated to work as partners in phosphorylating secretory proteins.^16^ The expression of FAM20A at a very late stage is consistent with the finding that inactivating this molecule in the dental epithelium did not cause significant morphologic changes in the tooth germ of the mandibular first molar at postnatal day 1. A high level of FAM20A was also observed in the stellate reticulum of the mandibular first molar in the 5-day-old mice and in the reduced enamel epithelium in eruptive-phase molars. Such expression profiling data provided invaluable clues regarding the biological roles of FAM20A in these tissues at different stages. In either the ameloblasts or odontoblasts, FAM20A protein is primarily located in the distal region of the cytoplasm towards the enamel matrix or dentin matrix. This unidirectional localization pattern of FAM20A on the secretory side of the cells supports the postulation that it is a Golgi-enriched protein colocalized with FAM20C.^16^

The μCT radiography analyses revealed a lack of true enamel in the molars of the 28-day-old cKO mice. Histologic evaluation showed that the molars of the cKO mice formed very little enamel matrix at postnatal day 5 or day 7, the ameloblast layer in the cKO mice was detached from the enamel matrix, and the ameloblasts in the cKO mice were low columnar or flattened, non-polarized and disorganized. The findings in the teeth of the cKO mice were consistent with the diagnosis of amelogenesis imperfecta (AI). The gross and radiographic appearance and histological changes in the enamel in the cKO mice were similar to those observed in mice with the constitutive ablation of *Fam20A*.^14^ The enamel defects, such as the scarcity of enamel matrix, detachment of the ameloblast layer from the enamel matrix and non-polarized/disorganized ameloblasts in the *Fam20A*-cKO mice, resembled those observed in the *Fam20C*-deficient mice;^12^ these similarities between the *Fam20A*-cKO and *Fam20C*-deficient mice supported the conclusions drawn from *in vitro* studies that FAM20A is required for the kinase function of FAM20C in phosphorylating certain enamel matrix proteins.^16^ Enamelin (ENAM) is believed to be a substrate of FAM20C, and the phosphorylation of this enamel matrix protein by FAM20C requires the presence of FAM20A.^16^ Without FAM20A, ENAM might not be properly phosphorylated, which can subsequently lead to the loss of ENAM function. When ENAM cannot be phosphorylated, its degradation is accelerated, which can lead to a reduction in its protein level. When ENAM cannot be properly phosphorylated, it might not be transported to the correct site, which could have led to the relatively diffused distribution pattern observed in the cKO mice, in contrast with the normal mice, in which ENAM was concentrated in the distal region of the ameloblasts close to the basal lamina zone. Like the *Fam20A*-cKO mice, *Enam*-null mice also showed AI phenotypes.^25, 26, 27^ Thus, the lack of proper phosphorylation in and/or the reduction of ENAM might be among the contributing factors causing the enamel defects in the *Fam20A*-cKO mice and in human patients with AIGFS and ERS. Further studies are warranted to examine whether the phosphorylation of enamel matrix proteins, including ENAM, is defective in cKO mice.

Tooth eruption delay associated with inactivating mutations in the *FAM20A* gene has been described in human patients^20^ but has never been reported in animal studies. Three components are essential to the eruption of teeth: reduced enamel epithelium, dental follicles and periodontal ligaments. When any of these three tissues is lost or dysfunctional, tooth eruption will be disturbed, causing lack of eruption or eruption delay.^28, 29^ In this study, the X-Gal and ISH analyses revealed a relatively high level of FAM20A in the reduced enamel epithelium in eruptive-phase molars, whereas this molecule was barely detectable in the dental follicles or periodontal ligaments. Thus, it is reasonable to postulate that FAM20A might be essential to the normal function of the reduced enamel epithelium in the control of tooth eruption. The tooth eruption delay might have hampered the masticatory function of the cKO mice shortly after they were weaned from their mothers, leading to mild malnutrition, which affected the growth of these mice for a short period of time. Thus, the cKO mice at postnatal 28 and 35 days had smaller body sizes, and subsequently, their body weights gradually caught up with those of the normal mice; after postnatal 35 days, the cKO mice might have gradually overcome the molar eruption delay and gained the proper mastication force required for chewing food and obtaining nutrition.

Gingival overgrowth has been observed in human patients suffering from AIGFS, but it has never been reported in animal studies. We observed severe gingival overgrowth in the cKO mice. The gingiva is made of two types of tissues: the epithelium on the surface and the connective tissue underlying the epithelium. It was unclear whether the gingival overgrowth in human patients is caused by abnormal proliferation of the epithelium or by hyperplasia of the connective tissue. That the *K14-Cre;Fam20A*^*flox/flox*^ mice, in which *Fam20A* is inactivated in the epithelium but is intact in the connective tissue, showed severe gingival epithelium overgrowth, which suggested that the gingival overgrowth in human patients suffering from AIGFS might occur primarily due to proliferation of the epithelium. The overgrowth of the gingival epithelium was not observed in *Fam20C*-deficient mice,^11, 12^ and it has not been reported in human Raine syndrome, which has been associated with *FAM20C* mutations.^6, 7, 8, 9, 10^ The observation that hyperplasia of the gingival epithelium was present in the *Fam20A*-cKO mice but not in the *FAM20C-*deficient subjects suggested that FAM20A might perform biological roles in this oral tissue independent of FAM20C, although *in vitro* studies have indicated that these two molecules might function in a dependent manner in phosphorylating certain secretory proteins.^16^ Further investigations are needed to elucidate the molecular mechanisms underlining hyperplasia of the gingival epithelium in *Fam20A*-deficient subjects.
