# Supplementary figures and images for: Loss of epithelial FAM20A in mice causes amelogenesis imperfecta, tooth eruption delay and gingival overgrowth
Source: Int J Oral Sci. 2016 Jun 3;8(2):98–109. doi: 10.1038/ijos.2016.14 (PMC4932772; doi:10.1038/ijos.2016.14)

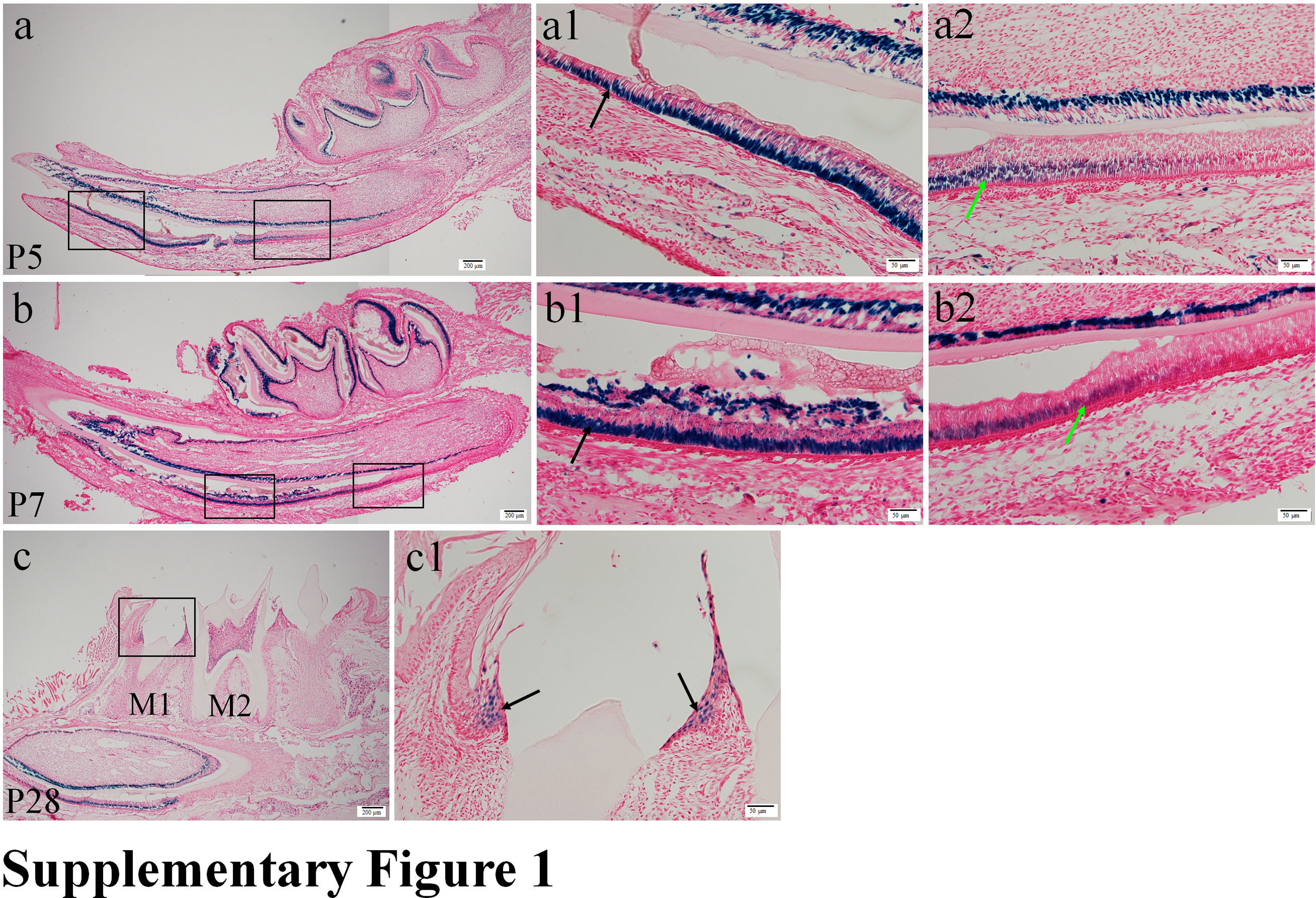

Supplement: Supplementary Figure 1 [file ijos201614x1.tif]
