# Supplementary material for: Loss of epithelial FAM20A in mice causes amelogenesis imperfecta, tooth eruption delay and gingival overgrowth
Source: Int J Oral Sci. 2016 Jun 3;8(2):98–109. doi: 10.1038/ijos.2016.14 (PMC4932772; doi:10.1038/ijos.2016.14)
Supplement: Supplementary Figure 1 Legend [file ijos201614x2.doc]

**Supplementary Figure 1. X-Gal staining in 5-, 7- and 28-day-old *Fam20A^lacZ-flox/+^* mice.**

(a) X-Gal staining of the mandibular incisor region from 5-day-old *Fam20A^lacZ-flox/+^* mice; (a1) higher magnification view of the left box area in (a); (a2) higher magnification view of the right box area in (a). (b) X-Gal staining of the mandibular incisor region from 7-day-old *Fam20A^lacZ-flox/+^* mice; (b1) higher magnification view of the left box area in (b); (b2) higher magnification view of the right box area in (b). (c) X-Gal staining of the mandibular molar region from 28-day-old *Fam20A^lacZ-flox/+^* mice; (c1) higher magnification view of the box area in (c). X-Gal staining revealed that, in the mandibular incisor, FAM20A signals were localized in the secretory stage ameloblasts (green arrows in a2 and b2) and maturation stage ameloblasts (black arrows in a1 and b1). FAM20A signals were present in the gingiva between the first molar (M1) and second molar (M2) of 28-day-old *Fam20A^lacZ-flox/+^* mice (c); in the gingiva, FAM20A was primarily localized in the epithelium (c1, black arrows). Bars in a, b and c = 200 µm; bars in a1, a2, b, b2 and c1 = 50 µm.
